# Metabolic Syndrome and Chronic Laryngitis: The Korean National Health and Nutrition Examination Survey 2008 to 2010

**DOI:** 10.1097/MD.0000000000001890

**Published:** 2015-10-30

**Authors:** Choung-Soo Kim, Seong-Soo Lee, Kyung-do Han, Young-Hoon Joo

**Affiliations:** From the Department of Otolaryngology-Head and Neck Surgery (C-SK, Y-HJ); Department of Endocrinology (S-SL); and Department of Biostatistics, College of Medicine, The Catholic University of Korea, Seoul, South Korea (K-DH).

## Abstract

Metabolic syndrome (MetS) is associated with a higher risk of morbidity and/or mortality for various chronic diseases. The aim of this study was to investigate the relationship of chronic laryngitis (CL) with MetS and its components in a representative Korean population.

Data from the Korean National Health and Nutrition Examination Survey (2008–2010) were analyzed. A total of 10,360 adults who had undergone otolaryngological examination were evaluated.

The prevalence of CL in the study population was 3.1%. The prevalence of MetS was significantly higher in patients with CL than in those without CL for both sexes (men: 34.7 ± 4.0% versus 25.9 ± 0.8%, *P* = 0.0235; women: 40.6 ± 5.3% versus 23.7 ± 0.7%, *P* = 0.0003). Elevated fasting glucose, triglycerides, and blood pressure, however, were only significantly associated with CL in women. After controlling for confounders, CL was only significantly associated with MetS in women (odds ratio: 2.159; 95% confidence interval: 1.2974, 3.594). Furthermore, the association between CL and MetS was most robust in women who were classified as obese.

In Korea, MetS and its components are significantly associated with CL in women.

## INTRODUCTION

Laryngitis generically refers to the inflammation of the larynx. Acute laryngitis is most commonly caused by a viral infection or strained vocal cords, and is typically self-limiting in less than 3 weeks.^1,2^ Chronic laryngitis (CL) is diagnosed when the signs and symptoms last for more than a few weeks, and usually requires treatment. Chronic laryngitis is most commonly caused by laryngopharyngeal reflux (LPR), poor laryngeal hygiene (including smoking and excessive alcohol intake), and some types of infection.^2–4^

Metabolic syndrome (MetS) is recognized as a cluster of metabolic risk factors that include abdominal obesity, dyslipidemia, hypertension, and hyperglycemia. It is associated with an increased risk of cardiovascular disease and type 2 diabetes. The prevalence of MetS has increased worldwide and also in Korea.^5,6^ Metabolic syndrome is strongly associated with an unhealthy lifestyle.^7,8^ Research suggests that a western-style diet, lack of physical activity, smoking, heavy alcohol consumption, and obesity are all risk factors for MetS.^9–14^ These risk factors may also be associated with CL.^3^ A relationship between CL and MetS, however, has not been investigated.

Therefore, the aim of this study was to investigate the common risk factors for CL and MetS, and to evaluate the relationship of CL with MetS and its individual components using data from the Korean National Health and Nutrition Examination Survey (KNHANES).

## PATIENTS AND METHODS

### Study Population

This study used data collected during 2008 to 2010 KNHANES (Korea Centers for Disease Control and Prevention). Conducted by the Division of Chronic Disease Surveillance under the Korea Centers for Disease Control and Prevention since 1998, KNHANES is a nationwide survey designed to accurately assess national health and nutrition. Field survey teams that included an otolaryngologist, an ophthalmologist, and nurses travelled nationwide in specially equipped mobile examination units. The survey consisted of a health interview, a nutritional questionnaire, and a physical examination. The KNHANES methodology has been described in detail previously.^15,16^

The study sample included 10,360 participants aged ≥19 years. Written informed consent was obtained from all participants before the survey. Approval for this study was obtained from the Institutional Review Board of the Catholic University of Korea, Seoul, South Korea.

### Survey for Chronic Laryngitis

A laryngeal examination was performed using a 4 mm, 70°-angled rigid endoscope with a charge-coupled device camera as described previously.^17^ Laryngoscopic findings of laryngitis and/or inflammation, including Reinke's edema, pseudosulcus, erythema, edema, or thick endolaryngeal mucus, were diagnosed as CL. Two otolaryngologic surgeons from the Korean Otolaryngologic Society verified video documentation and assessed the disease decision protocol. Video was obtained as 640 × 480-sized audio–video interleave files, which were compressed by DivX 4.12 codec using a compression rate of 6 Mb/s.

## LIFESTYLE

Information on medical history and lifestyle were collected using self-reported questionnaires. Smoking history was categorized into the 3 groups: current smoker, exsmoker, and nonsmoker. Subjects who drank >30 g/day were designated as “heavy drinkers.” Regular exercise was defined as strenuous physical activity performed for at least 20 minutes at least 3 times a week.

### Anthropometric and Laboratory Measurements

Weight and height were measured by well-trained medical professionals. Height was measured while standing; the subject faced directly ahead with shoes off, feet together, arms by the sides, and their heels, buttocks, and upper back were in contact with the wall. Height was measured to the nearest 0.1 cm using SECA 225 (Germany, SECA). Waist circumference (WC) was measured at the level of the midpoint between the iliac crest and the costal margin at the end of normal expiration to the nearest 0.1 cm. Weight was measured using a GL-6000–20 scale (Cass Korea) to the nearest 0.1 kg. Body mass index (BMI) was calculated as weight (kg)/height squared (m^2^). Based on the BMI, general obesity was defined as a BMI ≥ 25 kg/m^2^.^18,19^ Blood pressure (BP) was measured while subjects were in a sitting position following a 5-minute rest period. Systolic blood pressure (SBP) and diastolic blood pressure (DBP) were measured on the right arm using a mercury sphygmomanometer (Baumanometer, W.A. Baum Co., Copiague, NY). To assess the serum levels of biochemical markers, blood samples were obtained from the antecubital veins of the subjects following an overnight (10–12 hours) fast as described previously.^15^ Serum levels of fasting blood sugar, total cholesterol, triglycerides (TG), high-density lipoprotein (HDL) cholesterol, and low-density lipoprotein cholesterol were measured using an enzymatic method (Hitachi Automatic Analyzer 7600, Hitachi, Tokyo, Japan).

### Definition of Metabolic Syndrome

Metabolic syndrome was defined using the criteria proposed by the American Heart Association and the National Heart, Lung, and Blood Institute together with the International Diabetes Federation in 2009.^20^ Metabolic syndrome was defined as at least 3 of the following 5 components: a WC ≥ 90 cm in men and ≥80 cm in women, according to the International Diabetes Federation criteria for Asian countries; fasting glucose ≥100 mg/dL or receiving medication for elevated glucose; fasting TG ≥ 150 mg/dL (1.7 mmol/L) or a specific treatment for this lipid abnormality; HDL cholesterol <40 mg/dL in men and <50 mg/dL in women or receiving cholesterol lowering medication; and SBP ≥ 130 mm Hg and/or DBP ≥ 85 mm Hg or treatment of previously diagnosed hypertension.^20^

### Statistical Analysis

Statistical analyses were performed using the SAS survey procedure (version 9.3; SAS Institute, Cary, NC) to reflect the complex sampling design and sampling weights of KNHANES, and to provide nationally representative prevalence estimates as described previously.^15^ The procedures included unequal probabilities of selection, oversampling, and nonresponse in order that inferences could be made regarding adolescent participants.

Prevalence and 95% confidence intervals (CIs) for CL were calculated. In the univariate analysis, the Rao-Scott χ^2^ test (using PROC SURVEYFREQ in SAS) and logistic regression analysis (using PROC SURVEYLOGISTIC in SAS) were used to test the associations between CL and the risk factors in a complex sampling design. Participants’ characteristics were described using mean ± standard error for continuous variables, and number and percentage for categorical variables. Simple and multiple linear regression analyses were used to examine the association between CL and MetS. Analyses were adjusted for age, BMI, smoking status, alcohol intake, and physical activity (model 3). *P*-values were 2-tailed and *P* < 0.05 was considered significant.

## RESULTS

### General Characteristics of the Study Population

Among the 10,360 participants ≥19 years of age, 316 had CL. The prevalence of CL was 3.1% (3.7% in men and 2.4% in women). The baseline characteristics of the study subjects according to CL status are shown in Table 1. Subjects with CL were more likely to be older in both sexes. High SBP, DBP, and fasting blood sugar were significantly associated with CL in men. Mean BMI, SBP, DBP, and HDL cholesterol were significantly higher among women with CL than among women without CL.

**TABLE 1:**
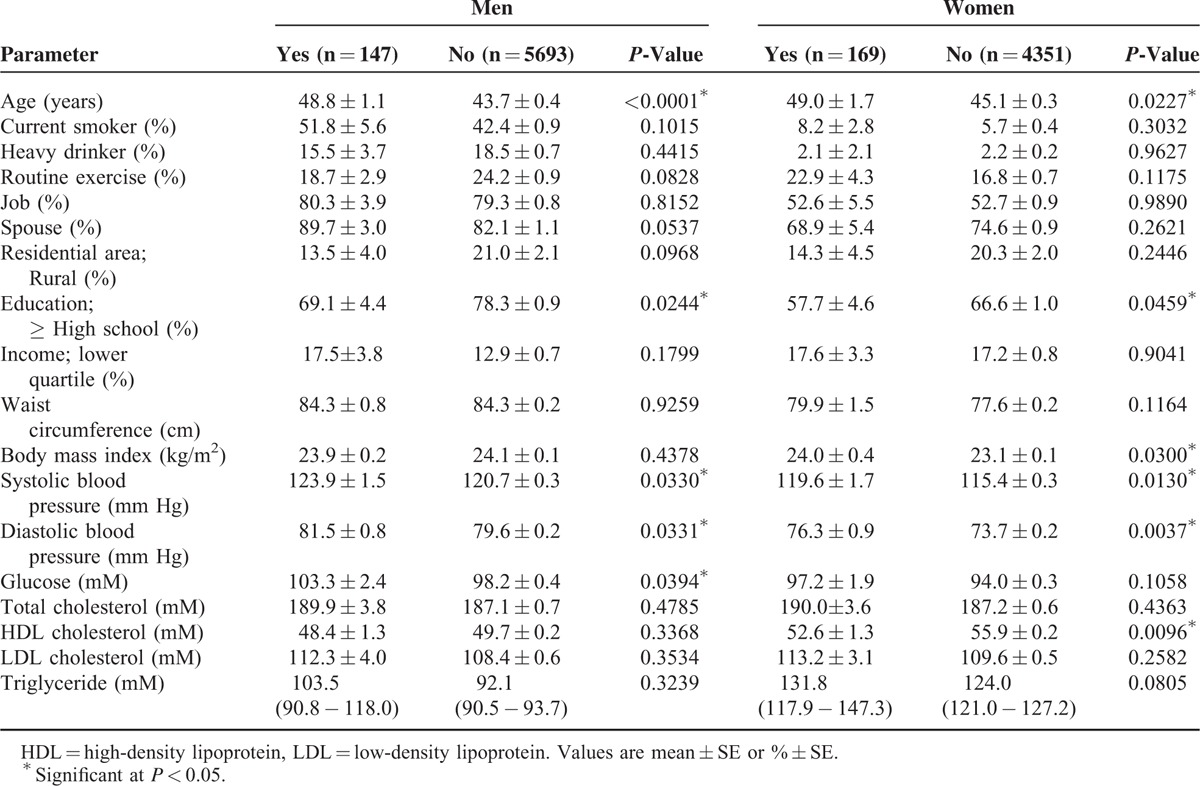
Analysis of Factors Potentially Associated With Chronic Laryngitis (n = 10,360)

### Prevalence of Chronic Laryngitis According to Metabolic Syndrome

**FIGURE 1:**
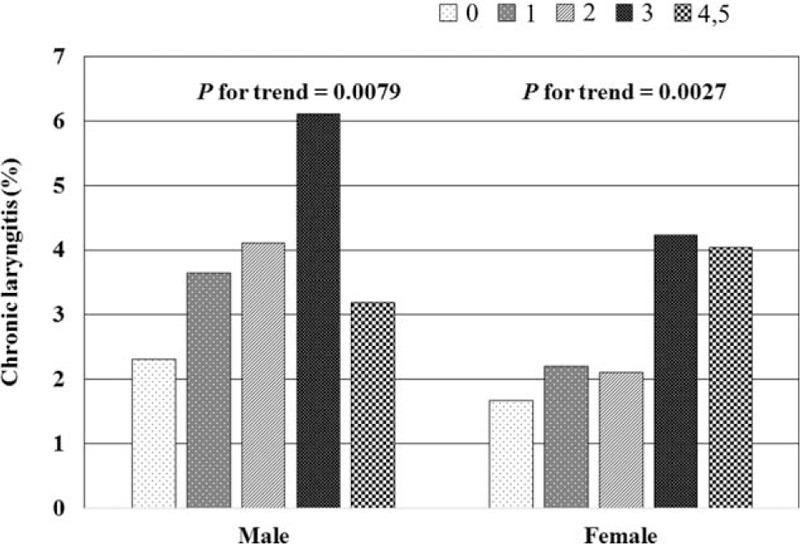
Prevalence of chronic laryngitis according to the number of metabolic syndrome components by sex.

The results indicated a sex difference in the relationship between the prevalence of CL and MetS components (Table 2). The prevalence of MetS was significantly higher in patients with CL than those without CL for both sexes (men: 34.7 ± 4.0% versus 25.9 ± 0.8%, *P* = 0.0235; women: 40.6 ± 5.3% versus 23.7 ± 0.7%, *P* = 0.0003). The prevalence of all MetS components, except WC, was higher in women with CL compared with those without CL. Only low HDL cholesterol was, however, found to be higher in men with CL. The prevalence of CL increased with an increase in the number of MetS components for both sexes (*P* for trend for men = 0.0079 and for women = 0.0027; Figure 1).

**TABLE 2:**
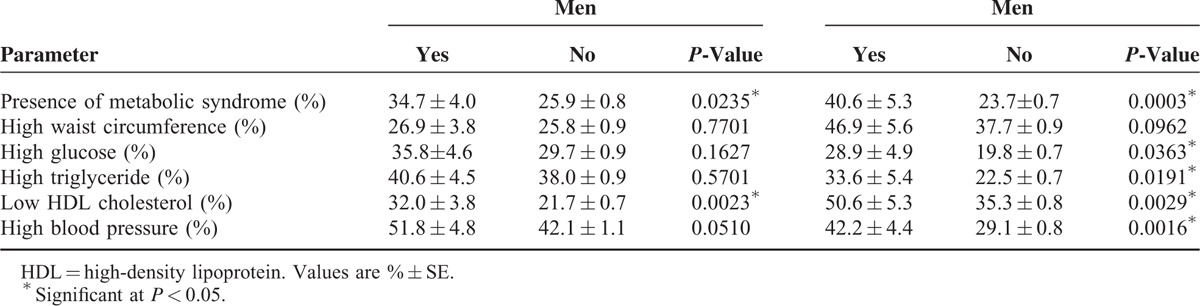
Prevalence of the Metabolic Syndrome Component According to the Presence or Absence of Chronic Laryngitis by Sex

**FIGURE 2:**
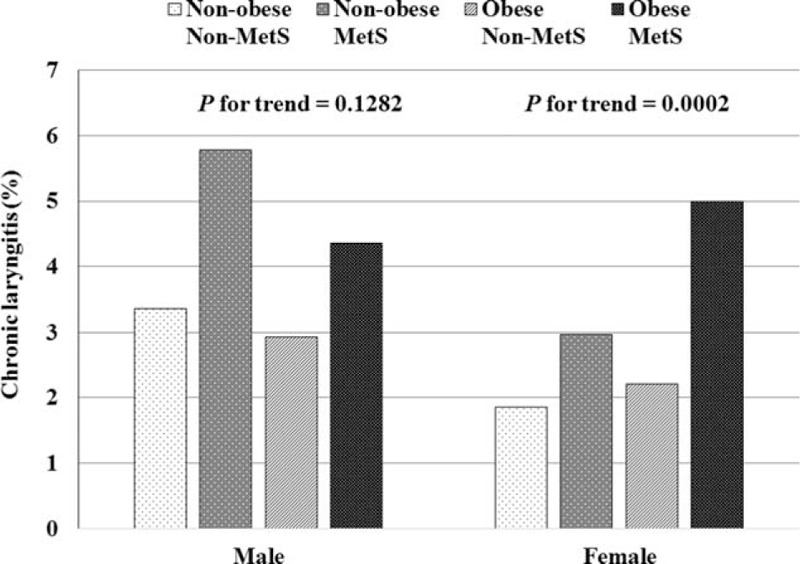
Prevalence of chronic laryngitis according to the 4 body composition subgroups.

Subjects were divided into 4 subgroups according to the presence or absence of obesity and MetS: nonobese nonMetS, nonobese MetS, obese nonMetS, and obese MetS. The overall prevalence of CL for women in the 4 subgroups was 1.86% for nonobese nonMetS, 2.97% for nonobese MetS, 2.21% for obese nonMetS, and 4.99% for obese MetS (*P* for trend = 0.0002; Figure 2).

### Multivariable Analyses of the Associations Between Chronic Laryngitis and Metabolic Syndrome

Table 3 shows the risk of developing CL according to MetS status for both sexes after adjustment for confounders. The adjusted odds ratio (OR) for CL was not significant for men with MetS. The risk of CL for women, however, was significantly associated with MetS [OR (95% CI): 2.032 (1.222, 3.380) in model 1, OR (95% CI): 1.878 (1.135, 3.107) in model 2, and OR (95% CI): 2.159 (1.2974, 3.594) in model 3], after adjusting for confounders.

**TABLE 3:**
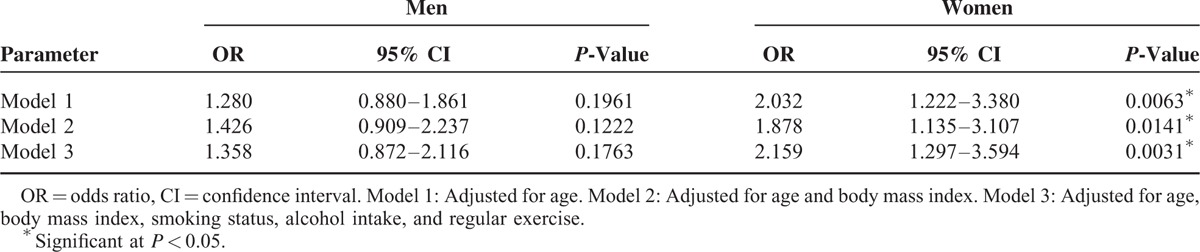
Logistic Regression Models of Metabolic Syndrome for Chronic Laryngitis

The risk of CL in the 4 subgroups was also calculated. After adjustment for age, BMI, smoking status, alcohol intake, and regular exercise, OR was 3.320 (95% CI: 1.507, 7.313) for obese women with MetS compared with nonobese women without MetS (Table 4). No significant association, however, was found in men.

**TABLE 4:**
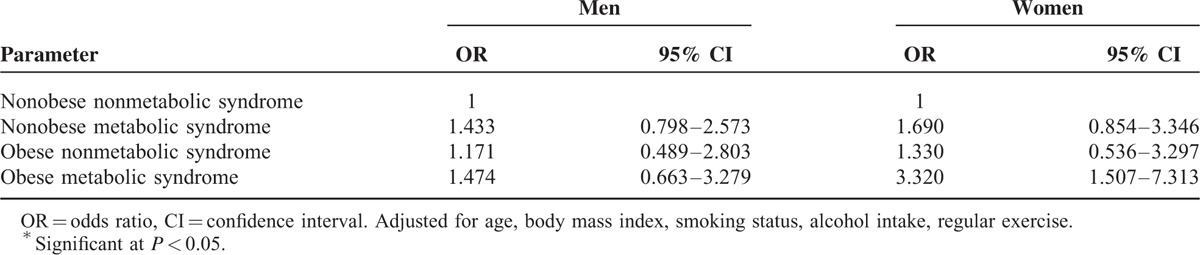
Adjusted Odds Ratio and 95% Confidence Intervals for Chronic Laryngitis According the 4 Composition Categories

## DISCUSSION

Chronic laryngitis usually develops gradually, and the underlying signs and symptoms can wax and wane during very long periods of time.^2–4^ Acute laryngitis is common, typically self-limiting, lasting a couple of weeks, and often associated with a purulent exudate. In CL, which is seldom considered an infectious process, the signs and symptoms of laryngitis last for months. Chronic laryngitis has an incidence of 3.5/1000 in the general population.^2^ Thus, as many as 21% of individuals will have CL in their lifetime. The prevalence of MetS is generally increasing, although the value varies according to the definition of MetS. According to the United States Department of Health and Human Services, the prevalence of MetS in the US was 35.1% for men and 32.6% for women in 2009.^18^ In Korea, the prevalence of MetS is lower than in the United States. According to KNHANES, the prevalence of MetS, however, increased from 24.9% in 1998 to 31.3% in 2007.^19,21^

To the best of our knowledge, this is the first large population-based study to examine the relationship of CL with MetS and its components among a representative Korean population stratified by sex. In this cross-sectional study, MetS and its components, including elevated fasting glucose, elevated TG, and elevated BP, were positively associated with the risk of CL in women. The risk of CL was significantly associated with MetS after adjusting for confounders. Furthermore, in the subgroup analysis, our results indicate that obese women with MetS have a higher prevalence of CL than nonobese women without MetS. No significant associations between MetS and CL, however, were found in men, regardless of the presence of obesity.

The reason for sex difference between MetS and CL is not clear but a few studies have reported associations of some components of MetS with CL among women. Litle et al reported that sex differences exist in both normal esophageal function and esophageal disease. In gastroesophageal reflux disease (GERD), there are sex differences in the pathophysiology and response to treatment.^22^ This sex-specific association also might be explained by differences of hormonal characteristics of MetS between men and women.^23^ And low levels of physical activity are strongly associated with the development of MetS and chronic diseases. Korean women with low levels of physical activity were at increased risk of MetS [OR (95% CI): 2.10 (1.15, 3.84)].^23^ In addition, there has been some evidence that sex difference in MetS contributes to the sex-related differential risk of cardiovascular disease and age-related cataract.^24,25^

Several possible mechanisms may explain how MetS and its components influence the development of CL. The signs and symptoms of CL can be associated with GERD, often referred to as laryngopharyngeal reflux or reflux laryngitis. Kallel et al^26^ showed that MetS was significantly more frequent in patients with GERD (50%) than in those without GERD (19.6%). Moreover, patients with MetS had a 2.82-fold increase risk to develop GERD as shown by multivariate analysis. Park et al^27^ found that MetS was more frequent in patients with esophagitis than in those without esophagitis (21 versus 13%; *P* < 0.001) with an adjusted odd ratio of 1.26. Previous studies have indicated that obesity can play a major role in CL. Excess body weight with a large WC produces higher intra-abdominal pressure, reducing esophageal sphincter pressure.^28,29^ A transient lower esophageal sphincter relaxation may be the most important reflux mechanism in obese subjects. Gastric distension leads to intense stimulation of both stretch and tension mechanoreceptors in the proximal stomach.^30,31^ High-TG levels were also associated with CL in this study. Several studies have revealed that a high level of serum TG is an important predictive factor for GERD or erosive esophagitis.^32,33^ Elevated BP was a risk factor for CL in this study. Hypertension has also been associated with GERD, after adjusting for BMI. Moki et al^34^ reported that hypertension was an independent risk factor for erosive esophagitis.

The current study has several limitations. First, there was no analysis about the relative severity or grade of the CL because objective testing and more detailed questions were not available. Second, the current study was cross sectional. We were unable to analyze the temporal association between CL and MetS. Third, the data were based on self-reports of several parameters, such as smoking, alcohol intake, and income, which may have led to underreporting and the possible introduction of some bias. Future studies employing prospective, randomized methods to examine CL are warranted.

In conclusion, data from KNHANES indicate that MetS and its components are associated with CL in women. The results of this population-based study on the prevalence of CL and its relationship with MetS will contribute to their prevention and management in the Korean population.
